# Modeling study of divertor particle and heat flux asymmetries for EAST H-mode discharges

**DOI:** 10.1038/s41598-022-16668-4

**Published:** 2022-07-27

**Authors:** G. Z. Deng, X. D. Lin

**Affiliations:** 1 Department of Radiotherapy, The First Affiliated Hospital of Hainan Medical University, Hainan Medical University, Haikou, 571199 China; 2 College of Physics and Optoelectronic Engineering, Shenzhen University, Shenzhen, 518060 China; 3 Advanced Energy Research Center, Shenzhen University, Shenzhen, 518060 China

**Keywords:** Nuclear physics, Plasma physics

## Abstract

The BOUT++ transport code is run to study the effects of plasma drifts on the divertor out-in asymmetries (DOIAs) of particle and heat fluxes and their decay widths for EAST lower single null H-mode discharges. The diamagnetic drift seems to have no effects on the DOIAs of total particle and heat fluxes due to its divergence-free nature. However, it could significantly increase the DOIAs of peak particle and heat fluxes and the flux decay widths. The E × B drift is found to induce a large plasma flow to the divertor region, enhancing the DOIAs of both total and peak particle and heat fluxes and the flux decay widths. Both the radial and poloidal components of the E × B drift are necessary in increasing the DOIAs, however, the radial E × B drift seems to play a more important role. The effects on the DOIAs caused by both diamagnetic and E × B drifts are reversed with the reverse of toroidal magnetic field. The heat flux decay width *λ*_q_ and spreading width *S*_q_ are important physical and engineering parameters for the divertors and could be obtained by fitting the heat flux profiles at divertor targets. The *λ*_q_ at the outer target from the simulation case with all drifts could well match with the multi-machine scaling proposed by Eich and the DOIA of *λ*_q_ is in reasonable agreement with the scaling proposed by Goldston.

## Introduction

The excessive particle and heat fluxes onto the plasma facing components (PFCs), especially the divertor targets, poses a severe threat to the lifespan of the divertor materials and the sustainability of the advanced steady state operation for tokamak fusion devices^1^. The particles and energy from the core plasmas along the magnetic field lines in the scrape-off layer (SOL) to the divertor, are usually found to be asymmetrically distributed between the outer and inner divertor targets^2^, which make the divertor particle and heat loads more of an issue to the tokamak fusion community. The DOIA is a general concept representing the unbalances of particles and energy at the outer and inner divertor targets. The larger DOIAs mean the differences of particles and energy between the outer and inner targets are larger. Understanding the DOIAs of particles and energy is of great importance to the design and operation of future high-power and long-pulse tokamaks like ITER and China Fusion Engineering Test Reactor (CFETR).

The studies of DOIAs have been carried out worldwide on present tokamaks such as DIII-D^3–5^, JET^6–8^, ASDEX-Upgrade^9,10^, LHD^11,12^, JT-60U^13,14^ and TCV^15^. The basic finding is the reversed asymmetric behavior with the reverse of toroidal magnetic field. The particle flux to the inner divertor target is usually larger than that of the outer divertor target for lower single null (LSN) discharges with normal toroidal magnetic field (ion B × ▽B direction towards the lower X-point), while the asymmetry is reversed with reversed toroidal magnetic field (ion B × ▽B direction directed away from the lower X-point). The DOIA of heat flux is usually opposite to that of particle flux^14^, resulting in a much larger heat flux to the inner or outer divertor target than the other one. A common explanation to the strong DOIAs of particle and heat fluxes is due to the existence of various types of plasma drifts, including the diamagnetic and E × B drifts^16–20^. Presently, the E × B drift has been widely recognized as the most important factor in enhancing the DOIAs. However, there is still dispute over which component (radial or poloidal) of the E × B drift playing the leading role. Rozhansky et al. presented that the poloidal E × B drift was the main factor in enhancing the DOIAs of plasma density and particle flux based on the study of sheath boundary condition at targets^18,19^. However, Chankin found that the radial E × B drift was playing the dominant role by analyzing the convective flows caused by the poloidal and radial components of the E × B drift based on a series of EDGE2D-EIRENE modeling^20^. It should be noted that most of the previous studies are focused on the DOIA of density or total heat flux without shedding light on the detailed distributions of particle and heat fluxes, especially the peak flux and the flux decay widths which are also of great importance to the safety and maintainability of target materials. Besides, although the diamagnetic drift is considered not to be important in inducing the DOIAs due to its divergence-free nature^21–23^, its effects on the DOIAs of target parameters have not been widely discussed yet.

EAST is the first superconducting tokamak in China with ITER-like divertor and configuration. Experimental studies on the DOIA of heat flux for the L-mode discharges^24–26^ and DOIA of particle flux for the H-mode discharges^27,28^ have been carried out recently. The results also show that the DOIAs could be reversed by reversing the toroidal magnetic field. The simulations of EAST discharges with SOLPS also demonstrate similar asymmetric behaviors with the experiment^29–31^. However, these modeling studies are mainly focused on the qualitative agreement between the simulations and the experiment without much analysis on the quantitative level. There is still a lack of effort on the effects of different types of drifts on the detailed distributions of particle and heat fluxes at the outer and inner divertor targets, especially the peak flux and the flux decay widths (including the particle and heat flux decay widths $\lambda_{js}$ and $\lambda_{q}$, and the particle and heat spreading widths $S_{js}$ and $S_{q}$). The understanding of these physics will be of special significance to the future high-power and long-pulse operation in EAST, which is the main focus of our studies in this paper.

The rest of the paper is organized as follows. Section Simulation setups briefly introduces the simulation setups of the BOUT++ edge plasma transport code. The effects of different types of drifts on the DOIAs of particle and heat fluxes and their decay widths will be discussed in detail in section Effects of drifts on the DOIAs of particle and heat fluxes and their decay widths. Finally, all the results will be summarized in section Summary and conclusions.

## Simulation setups

The work in this paper is carried out by the BOUT++ transport code (BTC). BOUT++ is a framework for implementing 2D and 3D plasma/fluid simulation in curvilinear geometry^32,33^, many physical models have been developed in this framework and the transport model is one of them^34–36^. The BTC is a 2D fluid model with drifts and neutrals. Starting from the Braginskii equations, the BTC includes evolutions of plasma ion density (${N}_{i}$), ion and electron temperature (${T}_{i}$ and ${T}_{e}$ respectively), parallel ion velocity (${V}_{\parallel i}$), electrostatic potential ($\phi$), vorticity ($\varpi$) and neutral density (${N}_{n}$). The recycling boundary conditions are applied at the divertor targets and wall. The E × B drift velocity ${{\varvec{V}}}_{ExB}=\frac{1}{{B}_{0}}{{\varvec{b}}}_{0}\times \nabla \phi$ and the ion diamagnetic drift velocity ${{\varvec{V}}}_{dia}^{i}=\frac{1}{{{Z}_{i}{eN}_{i}B}_{0}}{{\varvec{b}}}_{0}\times \nabla {P}_{i}$. The E × B and diamagnetic drift terms in the model could be switched on/off by the corresponding switches. The BTC has been applied to the edge plasma simulations of discharges from tokamaks like DIII-D^34^, C-mod^36^ and EAST^34,35^ and so on. The good performance of the code has proved it to be an effective tool for tokamak edge plasma simulation. Detailed information about the BTC can be found in^35^. Note that the grid is simplified in the divertor region, which may not be a big issue since benchmarks among BTC, SOLPS and the experiment have already been done in previous work^34,35^.

**Figure 1:**
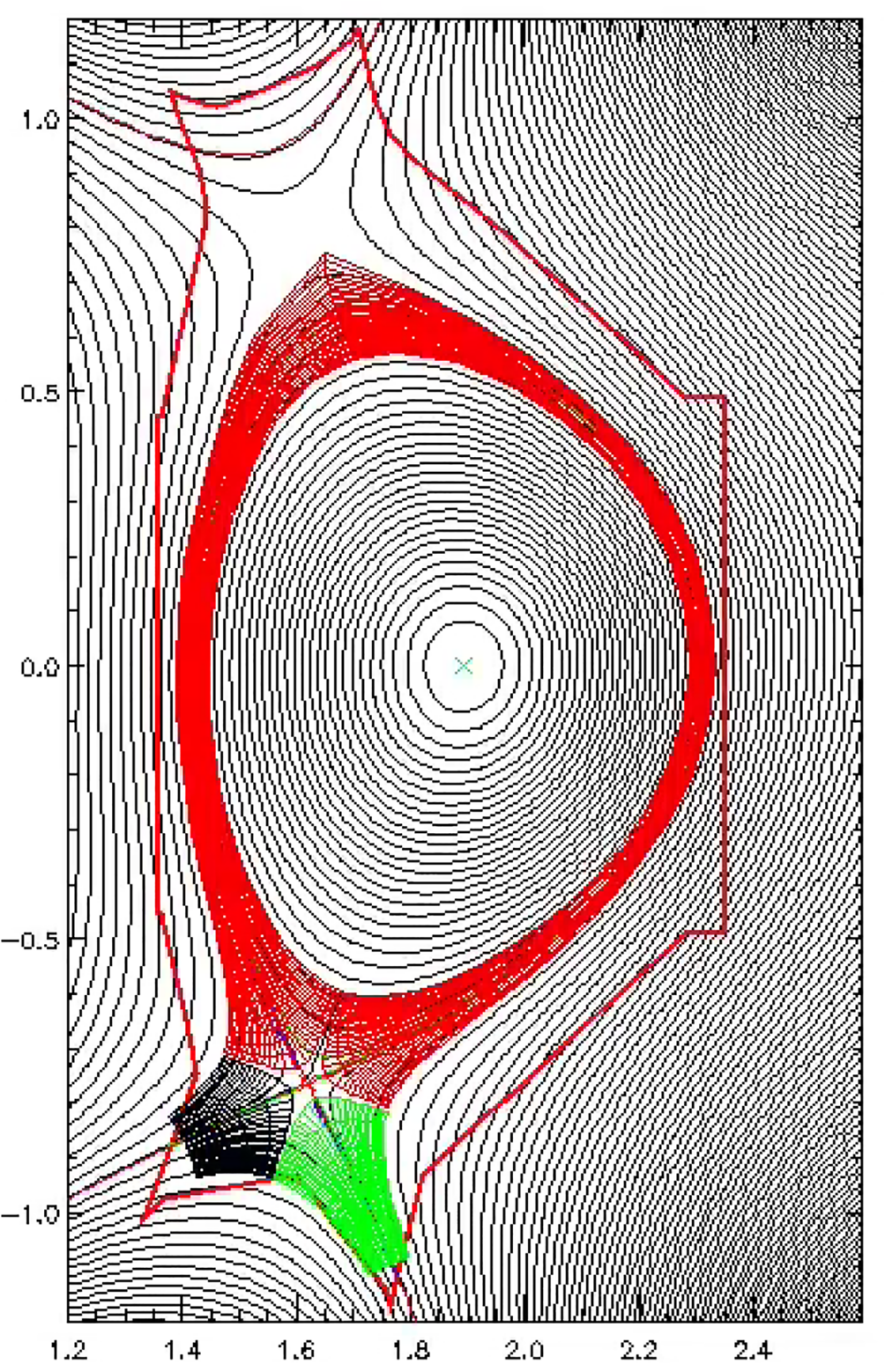
The grid image of BTC simulation for EAST shot #48,337 with a resolution of 36 × 64 (radial × poloidal).

**Figure 2:**
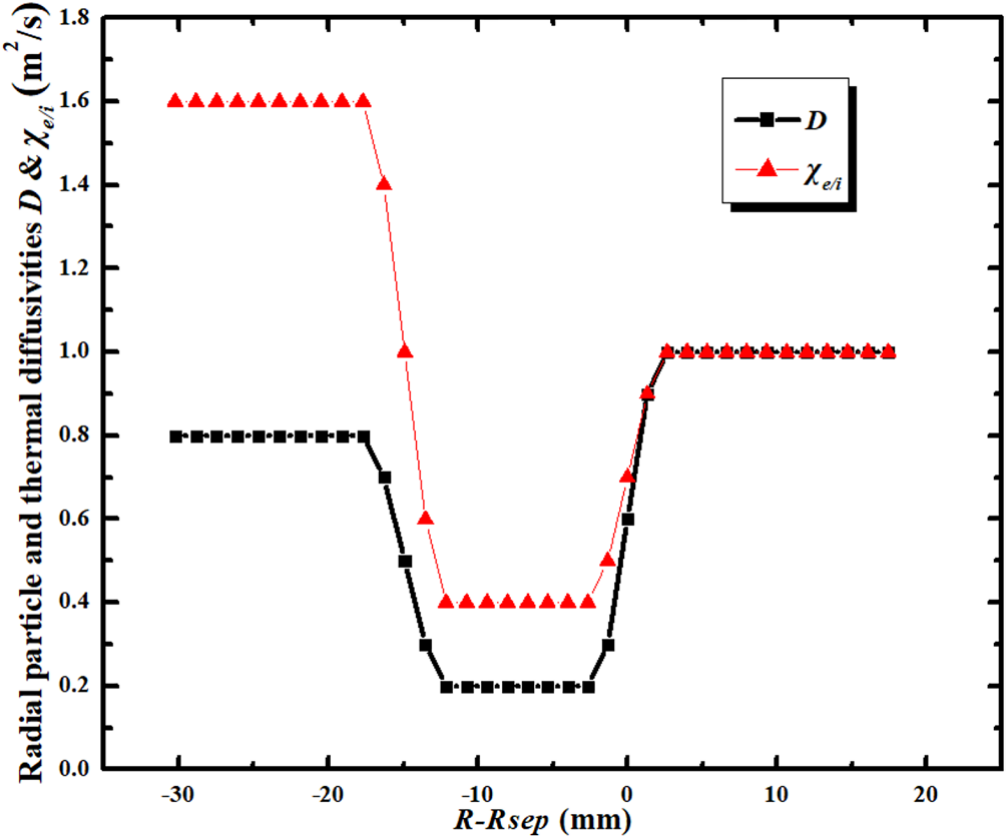
The radial plasma particle and thermal transport coefficient profiles for the BTC simulations of EAST discharge #48,337.

An EAST H-mode discharge #48,337 is chosen for the simulation. It is a LSN discharge with ion B × ▽B direction towards the X-point. The divertor is in a high-recycling regime for the discharge. The plasma current and toroidal magnetic field for this discharge are 0.4 MA and 2.2 T respectively. The simulation grid is generated based on the magnetic equilibrium of this discharge from the kinetic EFIT^37^ and is shown in Fig. 1. The plasma in the simulation is pure deuterium plasma with no impurities from seeding or sputtering at divertor targets. The density and temperature at core–edge interface (CEI) are fixed to be 2.8 × 10^19^ m^−3^ and 450 eV respectively. The “U” shaped profiles of transport coefficients are set in the simulation as shown in Fig. 2. The coefficients at the pedestal region are set to be small due to the existence of the transport barrier there, which is a common practice in edge plasma simulations^30,38,39^. The ion thermal diffusivity is set to be the same as the electron thermal diffusivity for simplicity. Note that the transport coefficients are by default poloidally constant in the simulation.

## Effects of drifts on the DOIAs of particle and heat fluxes and their decay widths

As aforementioned, the experimental studies of the DOIA of heat flux for L-mode discharges^24–26^ and the DOIA of particle flux for H-mode discharges^27,28^ have been carried out in EAST in recent years. There is still a lack of study on the DOIA of heat flux for EAST H-mode discharges due to the unreliability of heat flux measurement from the divertor Langmuir probes. For the H-mode discharges, since the heating power is relatively high, resulting in the arcing faults in some of the probe channels, which make the heat flux profiles from the divertor Langmuir probes unreliable. For the DOIA of particle flux for EAST H-mode discharges, the main finding is the reversed asymmetry behavior with the reverse of toroidal magnetic field^27^. In this section, we will study the effects of diamagnetic and E × B drifts on the DOIAs of particle and heat fluxes through modeling. The effect of toroidal magnetic field direction on the DOIAs will also be investigated for the same discharge (EAST #48,337) by artificially reversing the direction of toroidal magnetic field.

**Figure 3:**
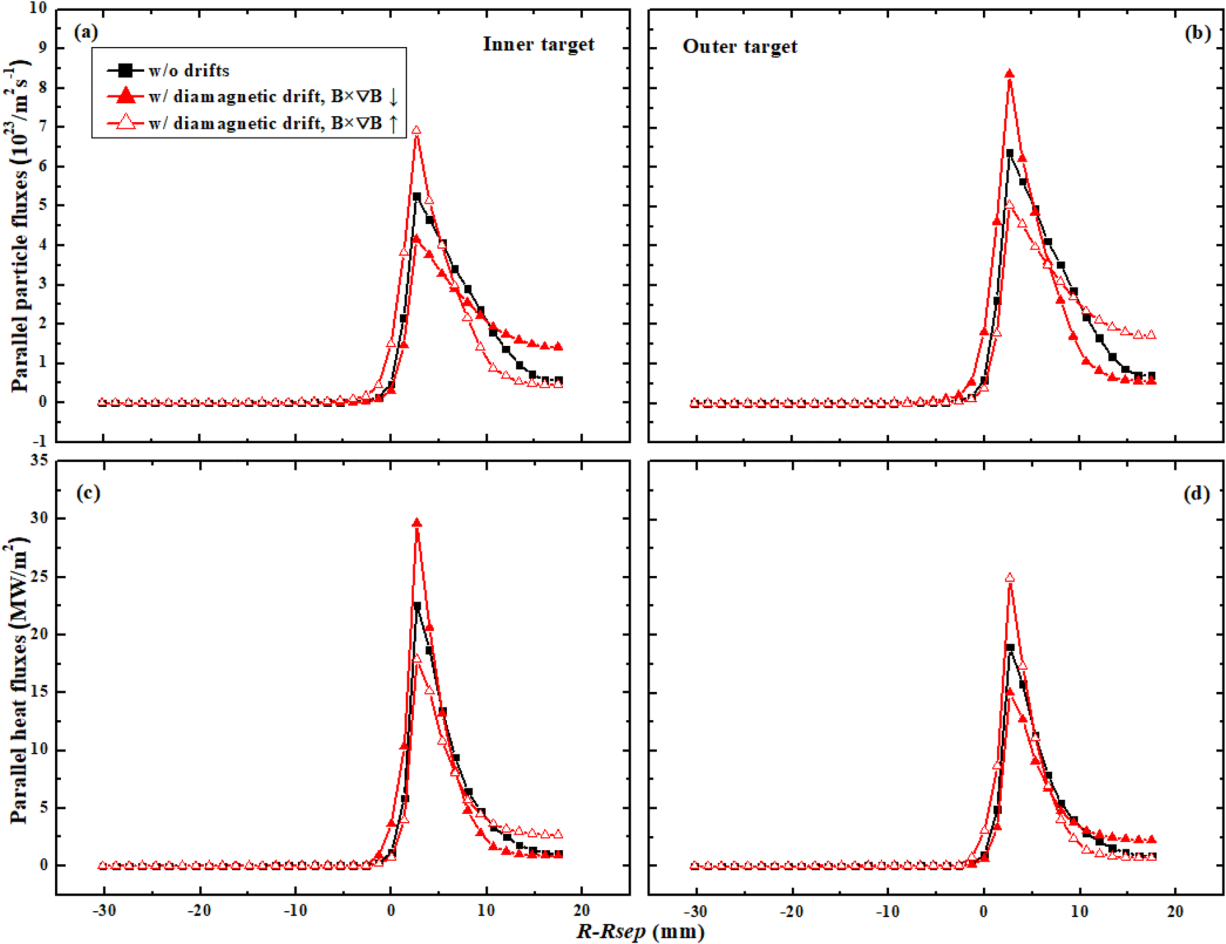
The divertor particle and heat fluxes at the inner and outer targets for the case without drifts and the cases with only diamagnetic drift.

The first set of simulations is carried out with only diamagnetic drift, and the simulation without drifts is also included to make comparison. The heat flux at one of the targets acts as an indicator to check whether a steady-state solution is achieved. Figure 3 shows the particle and heat fluxes from the simulation. The black squares and the red solid triangles represent the data from the case without drifts and with only diamagnetic drift under normal toroidal magnetic field respectively. The red hollow triangles represent the data from the case with only diamagnetic drift under reversed toroidal magnetic field. The abscissa coordinate is remapped to the outer mid-plane (OMP). As can be seen, the particle flux at the outer target is a little larger than that of the inner target for the case without drifts. The cross-sectional area at the low field side is larger than that of the high field side for the magnetic geometry, which may lead to a larger number of diffusion particles to the outer divertor target for the case without drifts. The DOIA of heat flux is opposite to that of particle flux. This is reasonable since the determination of heat flux relies more on the plasma temperature than the plasma density, and the increase of particle flux could result in the decrease of plasma temperature. Figure 3a,b show that the inclusion of diamagnetic drift could significantly change the distributions of particle fluxes at both targets. The peak particle flux decreases at the inner target while increases at the outer target for the case with normal toroidal magnetic field. The change of the particle flux decay width $\lambda_{js}$ seems to be opposite to that of peak particle flux. Both the peak heat flux and the heat flux decay width $\lambda_{q}$ in Fig. 3c,d show opposite trends with the counterparts of particle flux. The comparison between the case with normal toroidal magnetic field and the case with reversed toroidal magnetic field shows that the DOIAs of peak particle and heat fluxes and their flux decay widths are all reversed with the reverse of toroidal magnetic field.

**Figure 4:**
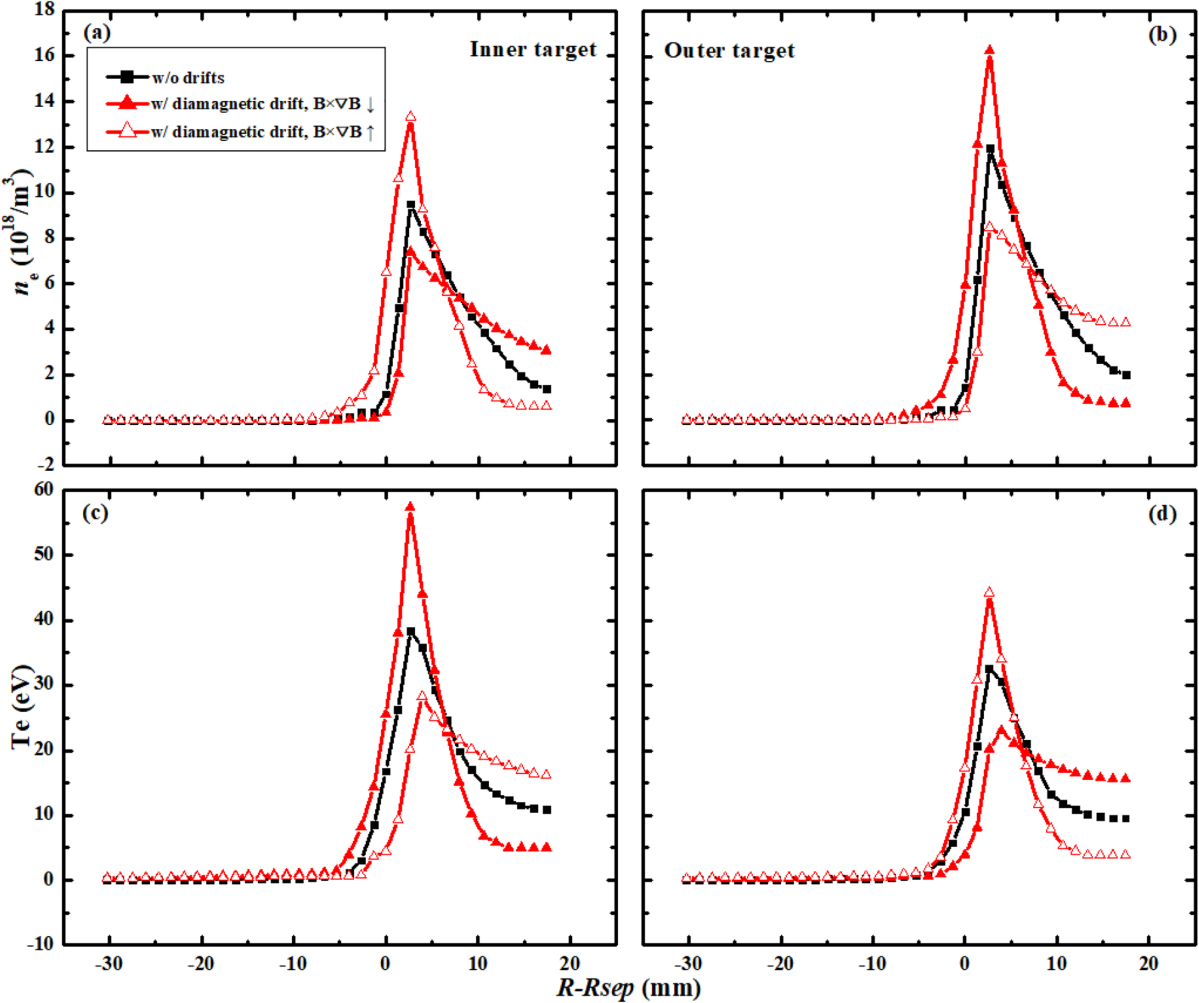
The divertor plasma densities and temperature at the inner and outer targets for case without drifts and cases with only diamagnetic drift.

**Figure 5:**
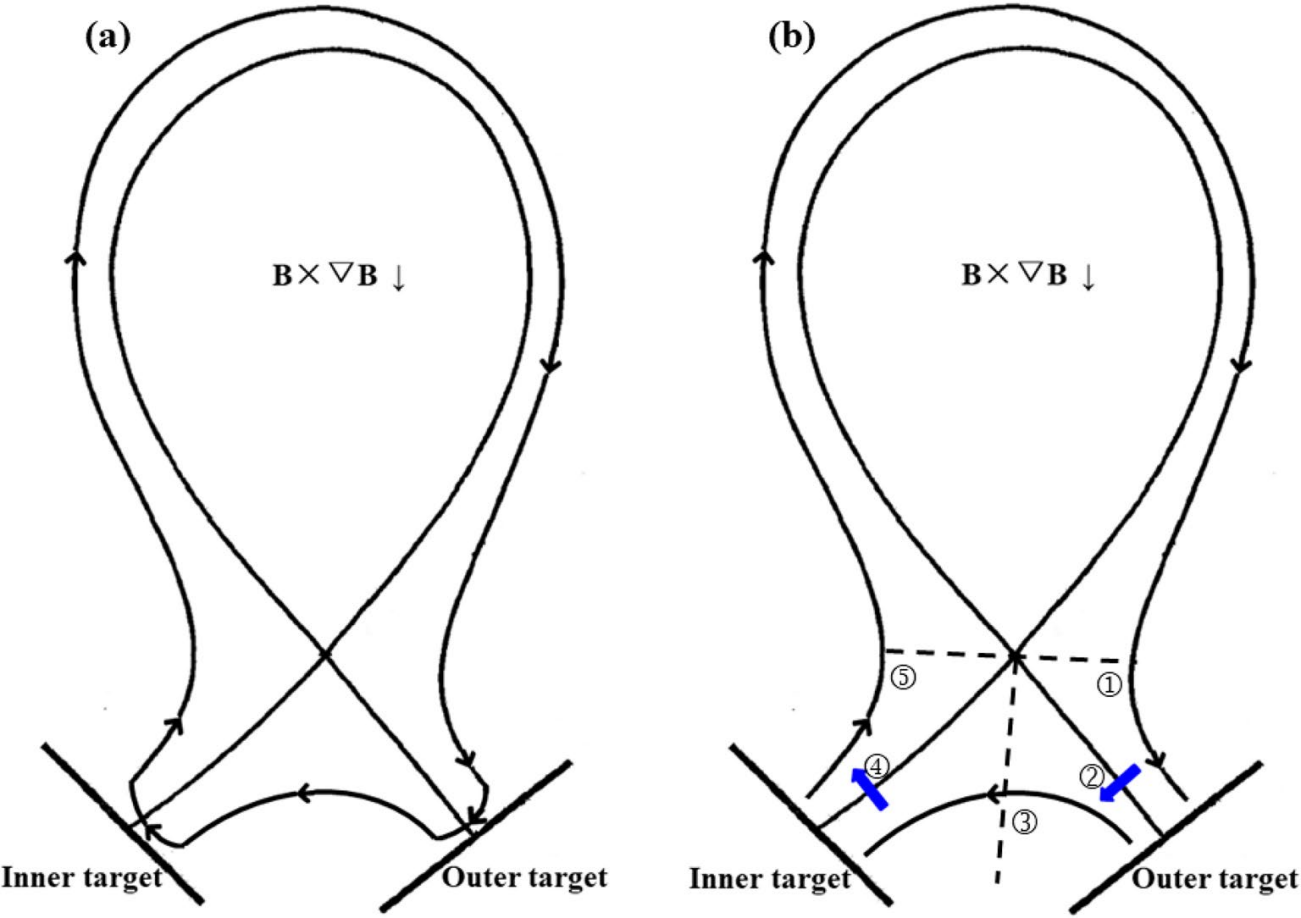
The diagrams of the directions of diamagnetic drift flow (left) and E × B drift flow (right) in the SOL and PFR regions for tokamak plasmas with normal toroidal magnetic field. In the right figure, the black arrows represent the direction of poloidal E × B drift flow while the blue arrows represent the direction of radial E × B drift flow. ➀: interface of the low field side upstream and the outer divertor region; ➁: interface of the SOL and PFR at the outer divertor region; ➂: interface of the outer and inner PFR region; ➃: interface of the SOL and PFR at the inner divertor region; ➄: interface of the high field side upstream and the inner divertor region.

Figure 4 shows the plasma densities and temperature for the simulation cases above. The plasma densities show similar trends with the particle fluxes in Fig. 3 for the three different cases which seem reasonable since more particle flux to the targets usually means larger plasma density at targets. The plasma temperature shown in Fig. 4c,d show opposite trends with the plasma densities, which result in the opposite trends of heat fluxes with the particle fluxes in Fig. 3. The left figure in Fig. 5 shows the diagram of diamagnetic drift flow direction in the SOL and private flux region (PFR) for tokamak plasmas with LSN configuration under normal toroidal magnetic field. The diamagnetic drift flows would change directions near the outer and inner targets, thus causing no net flows to the divertor surface^21–23^. In the low field side of the SOL, the diamagnetic drift velocity directs towards the outer divertor target, adding to the parallel particle transport from the upstream at the low field of the SOL to the outer target. Since the particle flux decay width is determined by the competition between the parallel and perpendicular transport, the larger parallel transport induced by diamagnetic drift could lead the larger peak particle flux and smaller particle flux width at the outer target as shown in Fig. 3b. On the contrary, the diamagnetic drift could lead to the decrease of peak particle flux and increase of particle flux decay width at the inner target. The diamagnetic drift flow direction is reversed with the reverse of toroidal magnetic field, which may have caused the opposite DOIA behaviors between the two simulation cases with opposite toroidal magnetic field directions. Table 1 shows the total particles and energy to inner and outer targets for the three cases above. The data show that the inclusion of diamagnetic drift does not change the total particles and energy to the targets, which is similar to the result in^21^ and has confirmed the divergence-free nature of diamagnetic drift. The numbers of particles across separatrix into the SOL induced by diamagnetic drift are also calculated for the two cases with diamagnetic drift. The results show that there are 6.37 × 10^17^ particles across separatrix into the SOL for the case with normal toroidal magnetic field. The number is −9.66 × 10^17^ for the case with reversed toroidal magnetic field. The numbers of particles across separatrix by diamagnetic drift for the two cases are more than 2 magnitudes smaller than the numbers of total particles to the divertor targets. The results above indicate that the diamagnetic drift could cause particles to cross flux surfaces and this process should be mainly happened in the divertor region. The diamagnetic current should be perpendicular to both the magnetic field lines and the pressure gradient. The pressure gradient in the parallel direction in the SOL couldn’t cause a diamagnetic current. However, the pressure gradient in the poloidal direction in the SOL should be able to cause a cross-field diamagnetic flow. In the upstream SOL, the pressure gradient along the poloidal direction in the SOL is very small, meaning that the cross-field particle transport induced by diamagnetic drift is weak, which may explain why the total particles and energy across separatrix due to the diamagnetic drift are small. In the downstream SOL of the divertor region, there is significant pressure loss in the poloidal direction, which causes a large pressure gradient and a large cross-field diamagnetic flow in the divertor region. This large cross-field flow leads the parallel flow induced by diamagnetic drift to change direction near the target, which results in almost no net diamagnetic flow to the target. Detailed analyses of this process are presented experimentally^21^ and numerically^22^ in previous literatures. Note that the total particles and energy to the outer boundary (wall) are much smaller than those of the targets for all of the three cases as shown in Table 1. In general, the simulation results here show that diamagnetic drift has almost no effects on the total particle and heat fluxes to the divertor targets. However, it could greatly affect the peak particle and heat flux and the flux decay widths at both divertor targets.

**Table 1:** Total particles and energy to the inner and outer divertor targets for case without drifts and cases with only diamagnetic drift.

	w/o drifts	w/diamagnetic drift, B × ▽B ↓	w/diamagnetic drift, B × ▽B ↑
Total particles at inner target	7.13 × 10^19^	7.25 × 10^19^	7.09 × 10^19^
Total particles at outer target	8.47 × 10^19^	8.44 × 10^19^	8.39 × 10^19^
Total particles at targets	1.56 × 10^20^	1.569 × 10^20^	1.548 × 10^20^
Total particles at wall	0.106 × 10^20^	0.101 × 10^20^	0.112 × 10^20^
Total energy at inner target	0.383 MW	0.368 MW	0.387 MW
Total energy at outer target	0.327 MW	0.335 MW	0.311 MW
Total energy at targets	0.71 MW	0.703 MW	0.698 MW
Total energy at wall	0.041 MW	0.055 MW	0.061 MW

**Figure 6:**
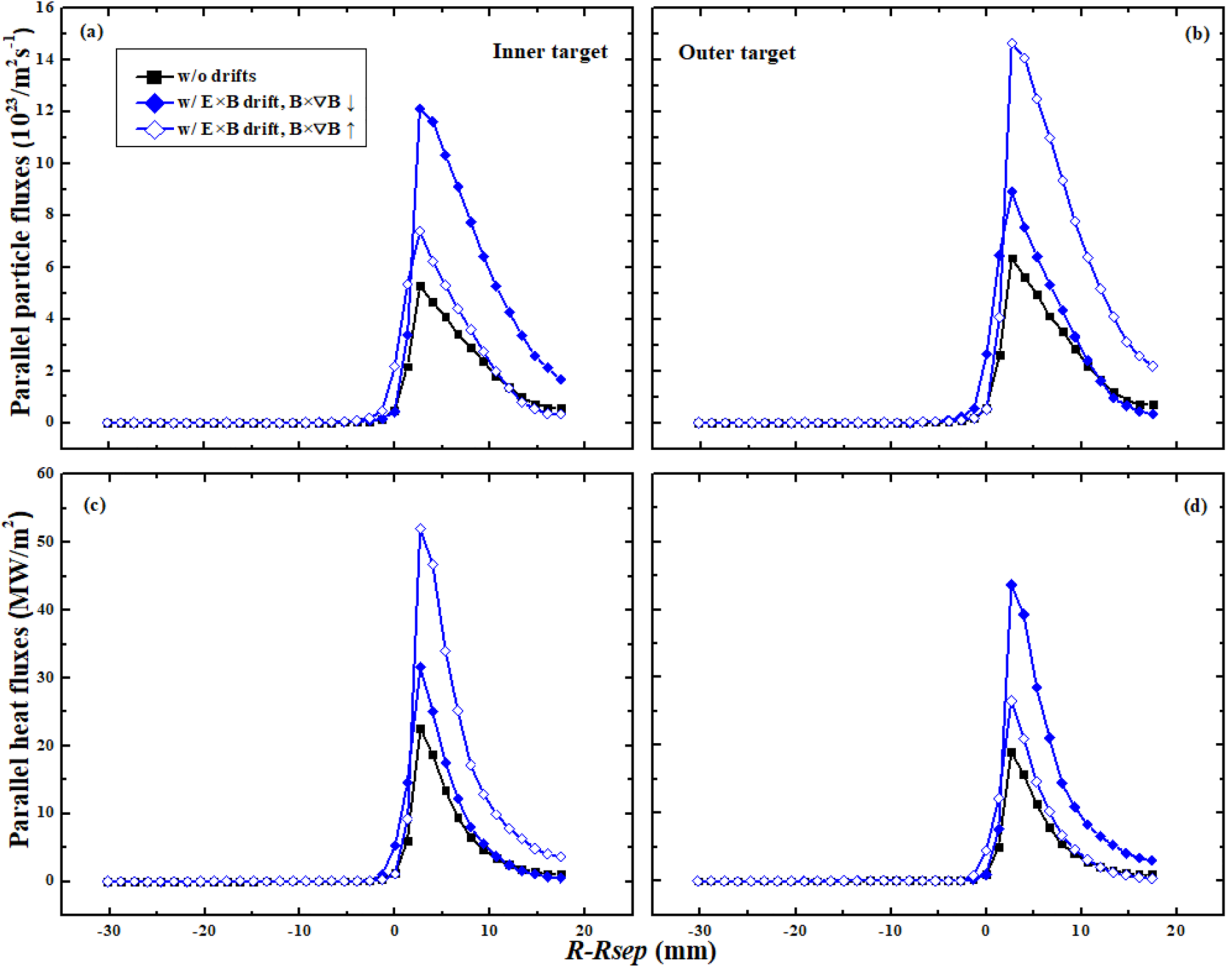
The divertor particle and heat fluxes at the inner and outer targets for case without drifts and cases with only E × B drift.

Another set of simulations is done with only E × B drift. Figure 6 shows the particle and heat fluxes at the inner and outer divertor targets from cases without drifts and with only E × B drift. As can be seen, the particle flux at the inner target increases significantly while it only slightly increases at the outer target for the simulation case with normal toroidal magnetic field when including the E × B drift. The heat flux however, significantly increases at the outer target while it only slightly increases at the inner target. The increases of particle and heat fluxes at both targets indicate that the E × B drift could induce net plasma flows from the upstream to the divertor targets. The right diagram in Fig. 5 shows the directions of E × B drift flows for tokamak plasmas with normal toroidal magnetic field. The black arrows represent the direction of poloidal E × B drift flow while the blue arrows represent the direction of radial E × B drift flow. The particles and energy are carried by the poloidal E × B drift flow from the upstream at low field side to the outer divertor region. Then these particles and energy are transferred to the inner divertor region by the radial E × B drift flow and the poloidal E × B drift flow in the PFR region. Some of the particles and energy return back to the upstream by the poloidal E × B drift flow at the high field side. The larger particle and heat fluxes at both targets after the inclusion of E × B drift indicate that the particles and energy across interface ➀ from the upstream at the low field side to the outer divertor region should be larger than those across interface ➄ from the inner divertor region to the upstream at high field side. The process of the DOIAs caused by E × B drift indicates that both the poloidal and the radial components of E × B drift should be necessary in impacting on the DOIAs of particle and heat fluxes. The significant differences of the increases of particle and heat fluxes between the inner and outer targets have caused strong DOIAs of peak and total particle and heat fluxes. The DOIAs of both particle and heat fluxes are reversed with the reverse of toroidal magnetic field, which is due to the reverse of the directions of E × B drift flows.

To study in detail on which component of the E × B drift plays the main role in inducing the strong DOIAs of particle and heat fluxes, the total particles across the interface ➀ to ➄ by E × B drift are calculated for the two simulation cases with different toroidal magnetic field directions and are shown in Table 2. The positive flow directions are shown in the right diagram of Fig. 5. For the case with normal toroidal magnetic field, most of the particles across interface ➀ into the divertor region return back to the upstream in the high field side by passing the interface ➄. However, a large number of particles are transported to the inner divertor region by the radial E × B drift and stay there, which eventually reach the inner target and cause the obvious DOIA of particle flux. The case with reversed toroidal magnetic field shows an opposite DOIA with respect to the case with normal toroidal magnetic field. The data show that both the poloidal and radial components of E × B drift are necessary in the determination of the DOIAs. However, the radial E × B drift plays a more important and direct role by carrying the particles from the inner/outer target to the other one. The numbers of particles across separatrix into the SOL induced by E × B drift are also calculated for the two cases with E × B drift. The results show that there are 1.81 × 10^20^ particles across separatrix into the SOL for the case with normal toroidal magnetic field. The number is 2.09 × 10^20^ for the case with reversed toroidal magnetic field. The numbers of particles across separatrix by E × B drift for the two cases are much larger than the counterparts that caused by the diamagnetic drift in previous simulation cases with diamagnetic drift.

**Table 2:** Total particles across the interface ➀ to ➄ by E × B drift for the two cases with different toroidal magnetic field directions.

Interface →	➀	➁	➂	➃	➄
w/ E × B drift, B × ▽B ↓	4.92 × 10^20^	4.47 × 10^20^	4.37 × 10^20^	4.31 × 10^20^	3.21 × 10^20^
w/ E × B drift, B × ▽B ↑	−3.16 × 10^20^	−4.62 × 10^20^	−4.76 × 10^20^	−4.85 × 10^20^	−5.32 × 10^20^

**Figure 7:**
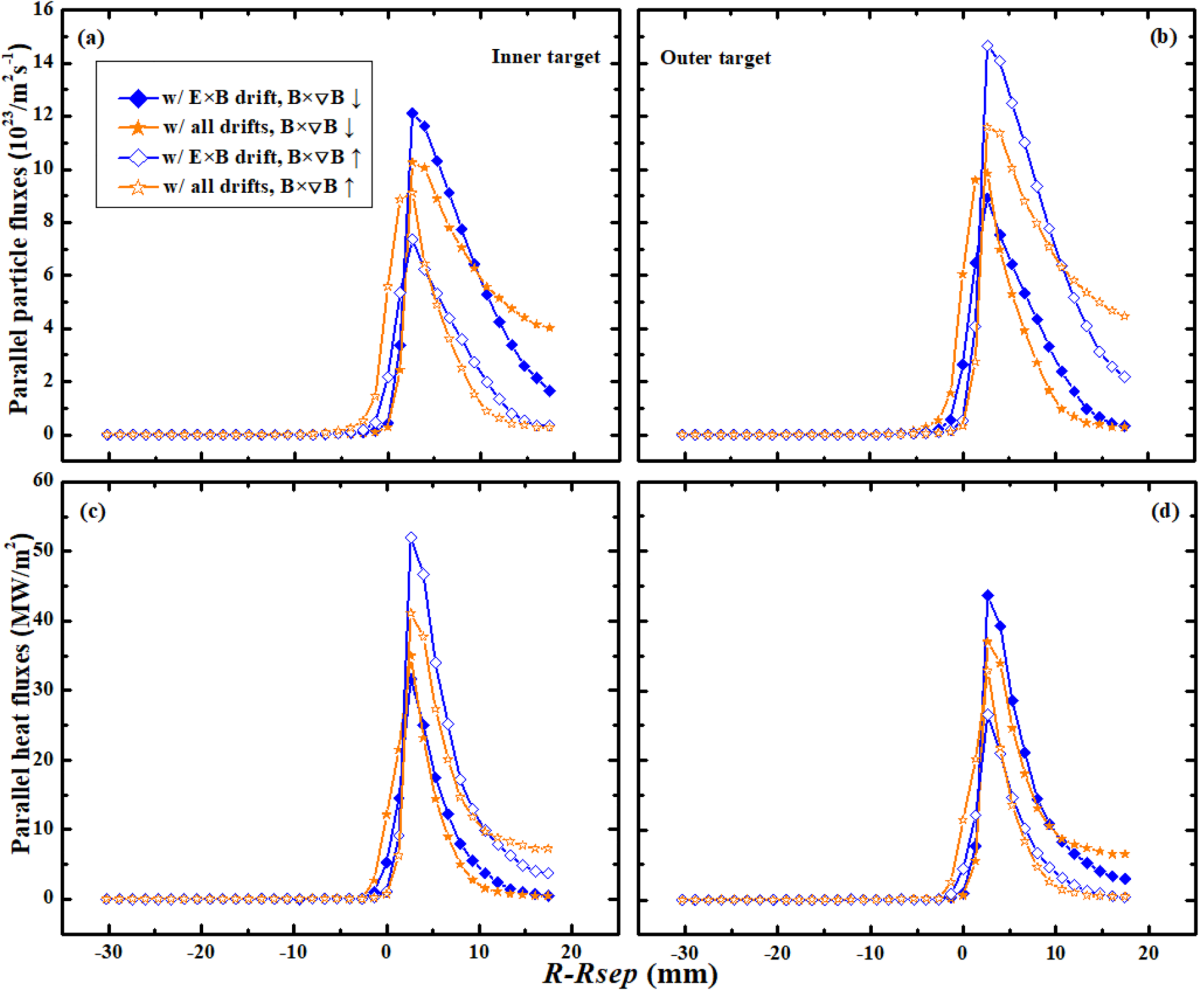
The particle and heat fluxes at the inner and outer targets for cases with only E × B drift and cases with all drifts under normal and reversed toroidal magnetic field.

The above simulations are done either with only diamagnetic drift or with only E × B drift. To study the effects of all drifts on the DOIAs of particle and heat fluxes, two simulation cases with normal and reversed toroidal magnetic field respectively, are done with all drifts switched on. Figure 7 shows the simulated particle and heat flux profiles at the inner and outer targets for the two cases along with the counterparts from cases with only E × B drift. For cases with normal toroidal magnetic field, the peak particle flux is smaller at the inner target while larger at the outer target in case with all drifts compared to that of with only E × B drift. The particle flux decay widths $\lambda_{js}$ at both inner and outer targets for the two cases seem to have opposite relationships compared with the peak particle flux. Meanwhile, the peak heat flux and the heat flux decay width $\lambda_{q}$ show reversed DOIAs with the counterparts of particle fluxes for the two cases. For the cases with reversed toroidal magnetic field, both the peak particle and heat flux and the flux decay widths between case with only E × B drift and case with all drifts have reversed relationships compared to those of the normal toroidal magnetic field. To study in detail on the DOIAs of particles and energy for these cases, the total particles and energy to the inner and outer divertor targets are calculated and shown in Table 3. As can be seen, the total particles and energy to both the inner and outer targets are larger in cases with only E × B drift than those of without drifts, indicating that the E × B drift could bring net particles and energy to the divertor targets, which is different from the diamagnetic drift. The total particles and energy to the inner and outer targets for the cases with all drifts are similar to the counterparts from the cases with only E × B drift, indicating that diamagnetic drift doesn’t have much effect on the DOIAs of total particles and energy, which has also been confirmed in the comparison between cases without drifts and with only diamagnetic drift. The data in Table 3 also show that the DOIAs of total particles and energy are reversed with the reverse of toroidal magnetic field, and the DOIA of energy is opposite to that of the particles for all the cases. The total particles and energy to the outer boundary are much smaller than those of the targets, which is similar with the results in Table 1.

**Table 3:** Total particles and energy to the inner and outer divertor targets for cases without drifts, with E × B drift and with all drifts.

	w/o drifts	w/ E × B drift, B × ▽B ↓	w/ E × B drift, B × ▽B ↑	w/ all drifts, B × ▽B ↓	w/ all drifts, B × ▽B ↑
Total particles at inner target	7.13 × 10^19^	1.97 × 10^20^	1.06 × 10^20^	2.02 × 10^20^	1.07 × 10^20^
Total particles at outer target	8.47 × 10^19^	1.23 × 10^20^	2.47 × 10^20^	1.2 × 10^20^	2.43 × 10^20^
Total particles at targets	1.56 × 10^20^	3.2 × 10^20^	3.53 × 10^20^	3.22 × 10^20^	3.5 × 10^20^
Total particles at wall	0.106 × 10^20^	0.186 × 10^20^	0.163 × 10^20^	0.191 × 10^20^	0.168 × 10^20^
Total energy at inner target	0.383 MW	0.624 MW	1.072 MW	0.611 MW	1.102 MW
Total energy at outer target	0.327 MW	0.914 MW	0.504 MW	0.921 MW	0.523 MW
Total energy at targets	0.71 MW	1.538 MW	1.576 MW	1.532 MW	1.625 MW
Total energy at wall	0.041 MW	0.089 MW	0.078 MW	0.085 MW	0.081 MW

**Figure 8:**
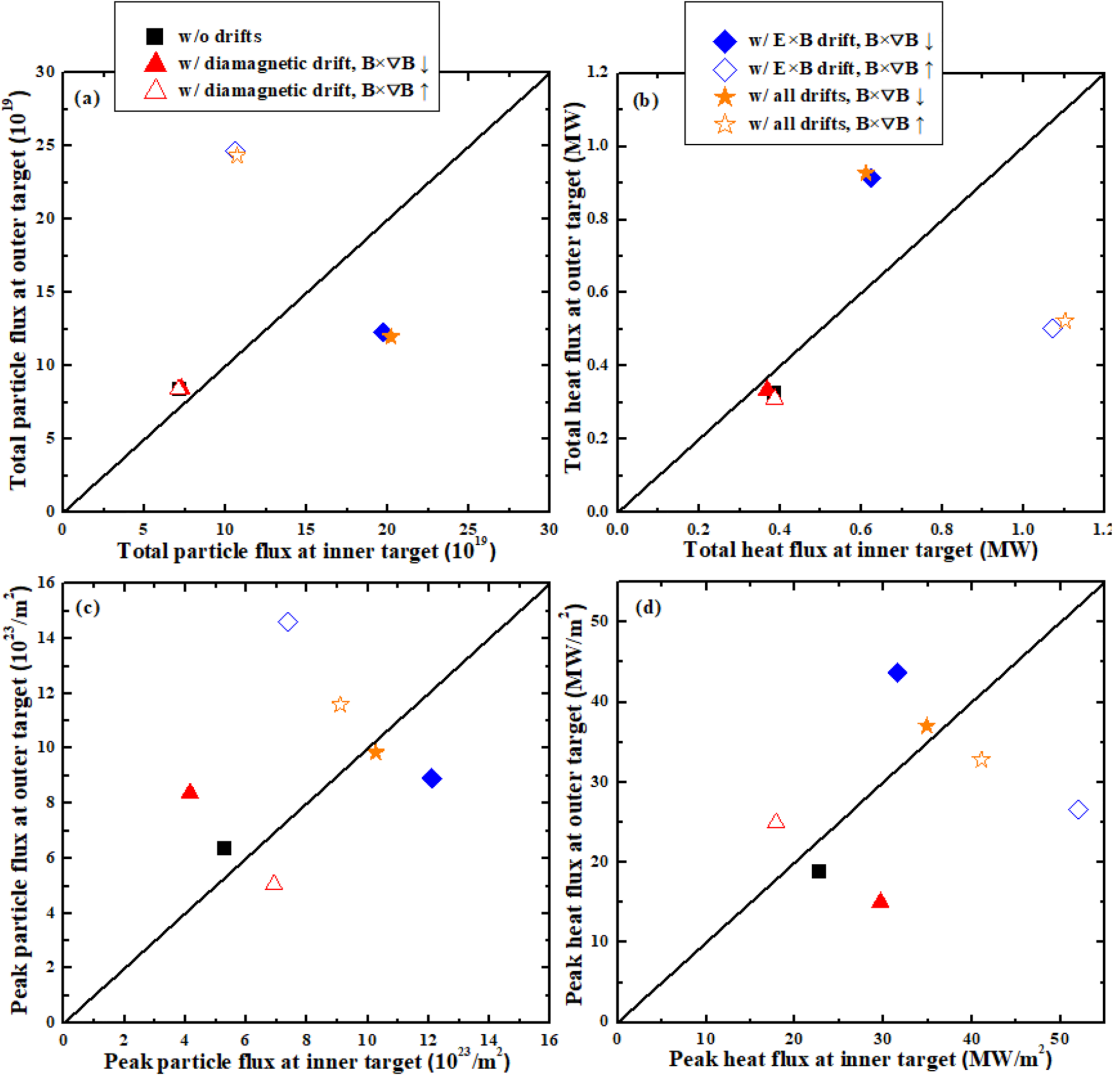
The total and peak particle and heat fluxes at the outer target versus the counterparts at the inner target for the cases without drifts, with only diamagnetic drift, with only E × B drift and with all drifts under normal and reversed toroidal magnetic field.

The above simulations show that the DOIAs of total particles and energy are mainly induced by the E × B drift while the diamagnetic drift almost has no effects on them. However, both the diamagnetic and the E × B drifts could affect the DOIAs of peak particle and heat flux and the flux decay widths. Figure 8 shows the total and peak particle and heat fluxes at the outer target versus the counterparts at the inner target for all of the above simulation cases. Figure 8a,b show that the diamagnetic drift has little effects on the DOIAs of total particles and energy and the asymmetries are mainly caused by the E × B drift. Figure 8c,d show that both the diamagnetic drift and the E × B drift could significantly affect the DOIAs of peak particle and heat flux. However, their effects are in the opposite ways. The joint effects from the diamagnetic drift and E × B drift for the cases with all drifts have led to the significant reductions of the DOIAs of peak particle and heat flux.

As we mentioned previously, both the diamagnetic and E × B drifts could significantly affect the particle and heat flux decay widths from the qualitative point of view. However, it is necessary to study the effects of both drifts on the flux decay widths in the quantitative point of view. The particle and heat flux decay widths could be obtained by fitting the particle and heat flux profiles at divertor targets using the fitting function proposed by Eich et al.^40^. Equation (9) is the fitting function which is a convolution of Gaussian at the strike point and exponential in the SOL region.

9 $$q(s) = \frac{{q_{0} }}{2}\exp \left( {\left( {\frac{S}{{2\lambda_{{}} }}} \right)^{2} - \frac{{s - s_{0} }}{{\lambda_{{}} f_{{\text{x}}} }}} \right) \cdot erfc\left( {\frac{S}{2\lambda } - \frac{{s - s_{0} }}{{Sf_{{\text{x}}} }}} \right) + q_{BG}$$

**Figure 9:**
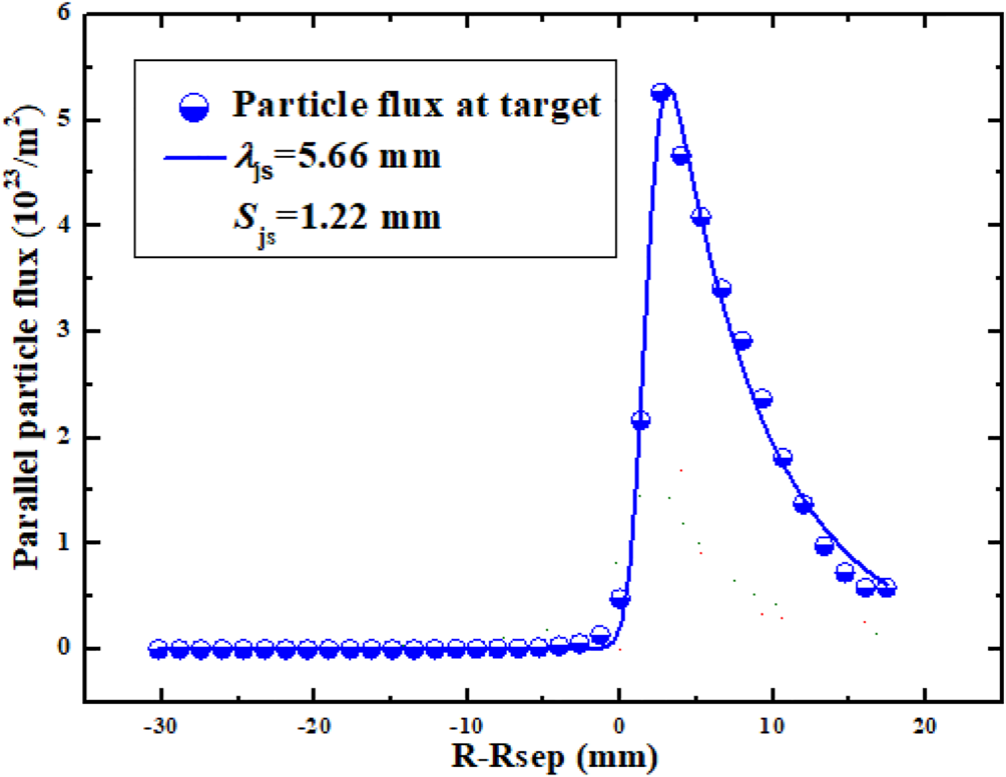
The parallel particle flux profile at the divertor target and the corresponding fit using Eq. (9).

*S* is the spreading width which characterizes the particle or heat dissipation into the PFR. $\lambda$ is the flux decay width mapped to the OMP. As particles and power are transported from OMP to the divertor targets, $\lambda$ is broadened by the magnetic flux expansion factor *f*_*x*_, which is defined to be the ratio of a specified extent at OMP to the extent at divertor target. $\overline{s} = s - s_{0}$, is the distance from a given target coordinate to the strike point *s*_*0*_. *q*_*BG*_ is the background flux intensity. Figure 9 shows an example of the fitting.

**Figure 10:**
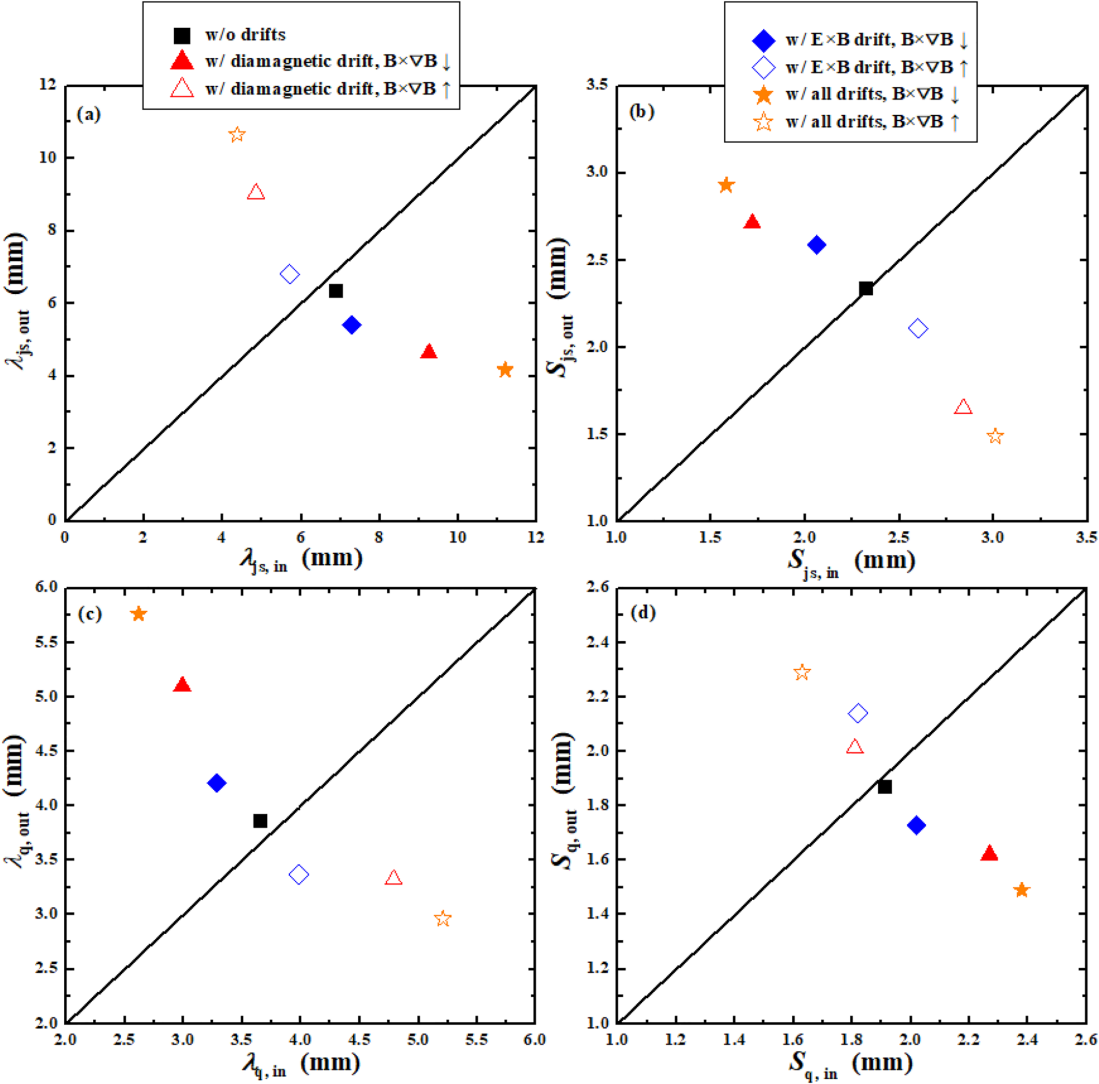
The particle and heat flux decay widths $\lambda_{js}$ and $\lambda_{q}$, and the particle and heat spreading widths $S_{js}$ and $S_{q}$ at the outer target, versus the counterparts at the inner target.

Figure 10 shows the particle and heat flux decay widths $\lambda_{js}$ and $\lambda_{q}$, and the particle and heat spreading widths $S_{js}$ and $S_{q}$ at the outer target, versus the counterparts at the inner target. As can be seen, the DOIAs of $\lambda$ and $S$ induced by diamagnetic drift for both particles and heat are much larger than those induced by the E × B drift. Both the diamagnetic and E × B drifts could lead to the increase of $\lambda_{js}$ and decrease of $S_{js}$ at the inner target for cases with normal toroidal magnetic field, while their effects on the $\lambda_{js}$ and $S_{js}$ at the outer target are in the opposite way. The DOIAs of $\lambda_{q}$ and $S_{q}$ are exactly opposite to those of $\lambda_{js}$ and $S_{js}$ for all the cases. The DOIAs of $\lambda$ and $S$ are reversed with the reverse of toroidal magnetic field. The solid and hollow stars in Fig. 10 show that the joint effects of diamagnetic and E × B drifts could lead to much larger DOIAs of the $\lambda$ and $S$ than that of with diamagnetic drift or E × B drift only.

**Figure 11:**
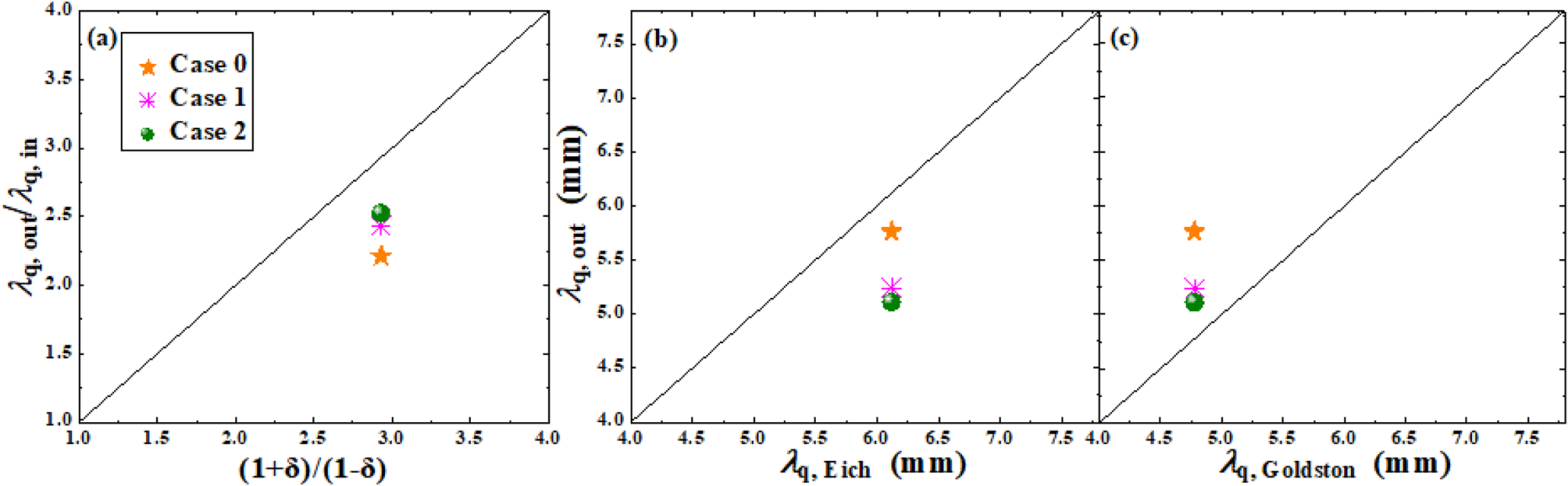
(**a**) The ratio of the outer $\lambda_{q}$ to the inner one for the simulation case with all drifts under normal toroidal magnetic field, versus the value predicted by the Goldston’s model. (**b**) and (**c**): the $\lambda_{q}$ at the outer target in the case with all drifts under normal toroidal magnetic field, versus the $\lambda_{q}$ predicted by Eich’s multi-machine scaling and Goldston’s drift-based model.

Since the particle and heat flux profiles measured by divertor Langmuir probes for this discharge on EAST are not smooth enough to carry out the fitting for the flux decay widths and spreading widths, it is hard to make comparisons of DOIAs of flux decay widths and spreading widths between the experiment and simulation. However, Goldston has proposed a drift-based model showing that the ratio of $\lambda_{q}$ at the outer target to $\lambda_{q}$ at the inner target is supposed to be (1 + δ)/(1−δ) with δ being the triangularity for double-null (DN) discharges^41^. Eich et al. also proposed a multi-machine scaling law for the $\lambda_{q}$ at the outer divertor target for H-mode discharges^42^. These scaling laws could act as alternatives to compare with the above simulation results. Figure 11a shows the ratio of the outer $\lambda_{q}$ to the inner one for the simulation case with all drifts under normal toroidal magnetic field, versus the value predicted by Goldston’s model. Since Goldston’s model assumes that radial transport of particles and energy are driven solely by drifts, another two simulation cases are run with smaller radial transport coefficients. Case 0, 1 and 2 shown in Fig. 11a represent the simulated results from the original case, the original case with 1/10 of the radial transport coefficients and the original case with 1/100 of the radial transport coefficients respectively. As the background transport gets smaller with smaller radial transport coefficients, the drifts play a more important role, which results in a larger DOIA of $\lambda_{q}$. The simulated result gets closer to Goldston’s model with smaller transport coefficients, but there is still discrepancy between the result in case 2 and the model with a difference of 12.5%. Note that previous simulation with BTC that reproduced the Goldston’s result didn’t turn down the particle diffusivity^43^. The fact that we reproduce reasonable agreement with Goldston’s result when turning down both heat and particle diffusivities indicates that drifts may dominate the heat transport compared to diffusion. Figure 11b,c show the $\lambda_{q}$ at the outer target in the simulation case with all drifts under normal toroidal magnetic field, versus the $\lambda_{q}$ predicted by Eich’s multi-machine scaling and Goldston’s drift-based model. The orange star in Fig. 11b shows that the discrepancy between the simulated $\lambda_{q}$ and the multi-machine scaling is about 6.7%. The data in Fig. 11c show that the simulated $\lambda_{q}$ gets closer to Goldston’s model with smaller transport coefficients, which seems reasonable since the model assumes no background transport other than drifts. Note that Goldston’s scaling of the ratio of $\lambda_{q}$ at the outer target to that at the inner target in his paper refers only to the DN discharges while the discharge studied in this paper is a LSN discharge. We should mention that for the predicted values from Goldston’s model both in Fig. 11a,c, the radial transport is assumed to be solely contributed by drifts, which means the discrepancies in Fig. 11a,c may mainly be caused by the background transport. In general, the simulated results in Fig. 11 are in acceptable agreement with the drift-based model and the multi-machine scaling.

## Summary and conclusions

A comprehensive study of the effects of plasma drifts on the DOIAs of particle and heat fluxes and their decay widths is carried out for EAST H-mode discharges with the BTC. The diamagnetic drift is found to have almost no effects on the DOIAs of total particle and heat fluxes due to its divergence-free nature. While the E × B drift could significantly affect the total particles and energy to the inner and outer targets, thus changing the DOIAs of total particles and energy. For the simulation with normal toroidal magnetic field, the E × B drift could lead to much larger particle fluxes to the inner target than the outer one, while the DOIA of energy is opposite to that of the particles. Detailed analysis shows that both the poloidal and radial components of the E × B drift are necessary in inducing the DOIAs of total particles and energy, however, the radial E × B drift seems to play a more important role. Both the diamagnetic and E × B drifts could affect the DOIAs of peak particle and heat flux and the flux decay widths. For the simulation with normal toroidal magnetic field, the diamagnetic drift could lead to the increase of peak particle flux at the outer target and the decrease of peak particle flux at the inner target. $\lambda_{js}$ show opposite trends to the peak particle flux and the DOIAs of $\lambda_{q}$ and heat flux are opposite to those of particle flux. The E × B drift could lead to the increase of peak particle flux at the inner target and the decrease of peak particle flux at the outer target, which are opposite to the effects of diamagnetic drift. However, the DOIAs of $\lambda_{js}$ and $\lambda_{q}$ induced by E × B drift are similar to those of the diamagnetic drift. The DOIAs of total and peak particle and heat fluxes and their decay widths are reversed with the reverse of toroidal magnetic field. The comparisons between the simulated $\lambda_{q}$ and the Goldston and Eich’s scaling laws show an acceptable agreement with small levels of discrepancy.

In general, the paper focuses on the quantitative studies of the effects of drifts on the DOIAs of particle and heat fluxes and their decay widths. The results are in reasonable agreement with the related scaling laws. However, more efforts are still needed to figure out the effects of other factors such as ballooning-like transport, plasma geometric effects from the targets themselves and divertor plasma conditions e.g., on the DOIAs of particle and heat fluxes, which will be our next-step work.

## Data Availability

The datasets and analyses details are available from the corresponding author on reasonable request.
